# The adductor tubercle can be a radiographic landmark for joint line position determination: an anatomic-radiographic correlation study

**DOI:** 10.1186/s13018-019-1221-y

**Published:** 2019-06-25

**Authors:** Kuang-Ting Yeh, Ing-Ho Chen, Chen-Chie Wang, Wen-Tien Wu, Kuan-Lin Liu, Cheng-Huan Peng

**Affiliations:** 1 Department of Orthopedics, Hualien Tzu Chi Hospital, Buddhist Tzu Chi Medical Foundation, No. 707, Zhongyang Rd., Sec. 3, Hualien, 97002 Taiwan; 2 School of Medicine, Tzu Chi University, Hualien, 97004 Taiwan; 3 Department of Orthopedics, Taipei Tzu Chi Hospital, Buddhist Tzu Chi Medical Foundation, New Taipei City, 23142 Taiwan

**Keywords:** Joint line, Adductor tubercle, Landmark, Knee arthroplasty, Bone loss

## Abstract

**Background:**

The adductor tubercle (AT) has been used intraoperatively as a landmark to evaluate the joint line position in knee arthroplasty. The purpose of this study was to determine whether the AT could be clearly identified on radiographic imaging as well as if the AT to joint line distance could be accurately measured for use as a radiographic landmark.

**Methods:**

The distance from the AT to the joint line was measured on each of 78 knees during total knee arthroplasty. Next, the AT was marked with a metal marker for radiographic analysis. On the postoperative radiograph, the location of the AT was determined by tracing the metal marker. Subsequently, the radiographic joint line distance (RJLD) was measured and compared with the intraoperative joint line distance (IJLD) to test the agreement of the measurements.

**Results:**

Location analysis indicated that the inflection point on the radiographic contour of the distal femur was the predicted location for the AT. The mean IJLD was 45 ± 3 mm and the RJLD was 45 ± 4 mm. The intraclass correlation coefficient was used to evaluate the inter-rater reliability between the two methods; that coefficient was 0.751, indicating good agreement between them. Measurements on the radiograph were comparable to the intraoperative measurements of the operated knees.

**Conclusions:**

In addition to being an intraoperative landmark, the AT may also be an eligible radiographic landmark for analyzing joint line level. The RJLD measurement may be obtained to plan the joint line position in knees with significant bone loss preoperatively and to follow up the results of surgery postoperatively.

## Background

Restoration of the joint line position in knee arthroplasty is a critical goal for achieving improved kinematics, optimal ligamentous stability, patellar tracking, and the resulting optimal outcomes [1–6]. This practice is especially crucial for knees with significant bone defects, for which the appropriateness of the distal condylar reconstruction must be ensured [4, 7, 8]. Surgeons have employed various landmarks to determine the joint line position, such as the tibial tubercle, lower pole of the patella, tip of the fibular head, and medial epicondyle of the femur [4, 9–13].

Landmarks may be classified as intraoperative or radiographic according to their anatomical characteristics and how they are used. An intraoperative landmark can be used during surgery to assess the joint line position, whereas a radiographic landmark is clearly visible on a radiograph and helps determine the joint line level on preoperative or postoperative radiographs. An ideal landmark should function well both intraoperatively and radiographically. Hence, preoperative planning, intraoperative management, and postoperative follow-up can be integrated, resulting in best practice for the restoration of the joint line position. However, none of the aforementioned landmarks provide such dual functionality.

The adductor tubercle (AT) is a bony prominence on the medial surface of the medial condyle and has recently been found to be an intraoperative landmark [14–18]. Our previous study showed that the AT has the desirable anatomical features to be an ideal intraoperative landmark [18]. Moreover, it is located well away from the distal tibiofemoral joint and has the advantage of being less likely to be damaged in knee arthroplasty, which is associated with significant bone loss. Could the AT also be an eligible radiographic landmark? Although some authors have reported a radiographic evaluation of the joint line using the AT [5, 14, 15, 19, 20], we found their methods of AT localization to be inconsistent and even confusing. Thus, methods to identify the AT have not been described well, and the AT is not a proven radiographic landmark.

The purpose of this research was to conduct an anatomic study with radiographic correlation, to demonstrate that the AT can be a radiographic landmark as well as an intraoperative one. We sought to answer two questions: (1) where is the AT located on an anterior-posterior knee radiograph? (2) are the joint line distances of the intraoperative and radiographic measurements equal?

## Materials and methods

### Intraoperative study

Patients admitted to our university teaching hospital for primary total knee arthroplasty in the end stage of osteoarthritis were invited to participate. This study was conducted after obtaining approval from the Institutional Review Board of the hospital, and all subjects signed an informed consent form. No funding was received in this study. With the intent not to include knees with abnormal morphology, patients who presented with joints that had severe varus deformity (tibiofemoral angle > 15°) and previous surgeries or fractures over the femoral condyle were excluded. From January 2013 through December 2013, a total of 78 patients met these criteria and were included in the study; 66 were women and 12 were men, with a mean age of 71 years (range = 46–86 years).

One surgeon (IHC) performed all operations and intraoperative measurements. All patients received a posterior stabilized condylar knee prosthesis (U2; United Orthopedic Company, Taiwan) using a measured resection technique. The procedure was performed through a small incision with a midvastus arthrotomy [21]. Identification of the AT and measurements of the AT to joint line distance were performed once all prosthetic components had been cemented and the wound was about to be closed. At this moment, visualization of the landmark was optimal, obviating the need for additional dissection.

**Fig. 1:**
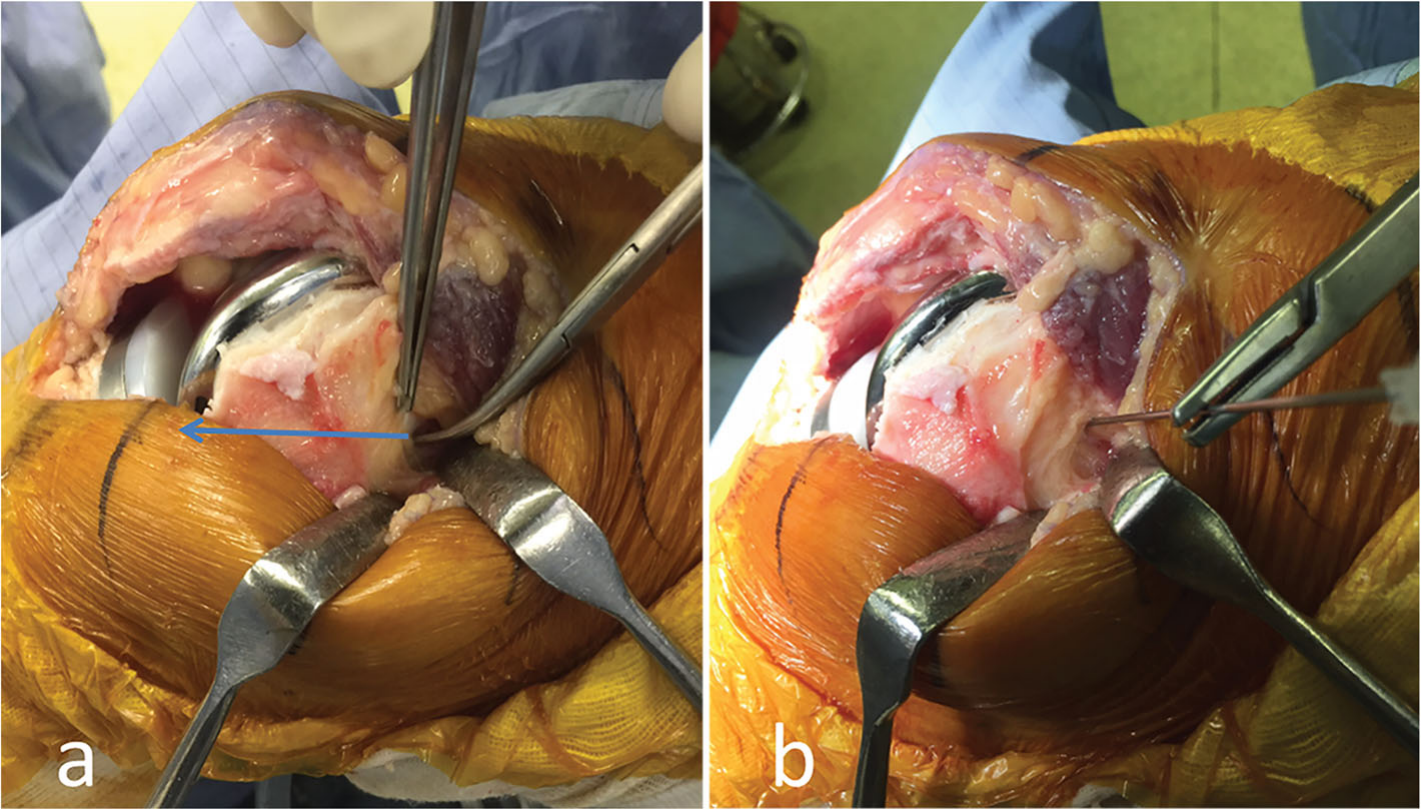
Our method of measurements. **a** Measurement of the intraoperative joint line distance (IJLD) on a knee undergoing total knee replacement. The nose of the hemostat indicates the adductor tubercle. The arrow line depicts the distance from this landmark to the distal condylar surface, or the IJLD, which is measured with a caliper. **b** Next, an 18-G spinal needle pinpoints the same landmark. A 2-mm-long segment of the metal stylet is tapped into the bony surface from within the sheath of the needle to mark the adductor tubercle for radiographic analysis

The AT is located in the most anterior part of the corner formed by the medial and superior surfaces of the medial condyle. Because the AT has little protrusion and is difficult to locate by fingertip palpation, we used the nose of a hemostat (another tipped instrument could be used) to pierce a thin layer of soft tissue covering the presumed AT location and struck the bony surface. We then slid the nose of the hemostat back and forth along the bony surface until it slipped into the superior surface of the medial condyle, which indicated that the AT had been engaged. When the adductor tubercle had been labeled with the nose of the hemostat, the distance from this structure to the most distal femoral condylar surface, concerning the longitudinal axis of the femur, was measured using a caliper. This distance was defined as the intraoperative joint line distance (IJLD) (Fig. 1a). All measurements were performed in triplicate, and the mean values were used in the analysis. Details of this technique can be found in our previous report [18].

Finally, a metal marker made of the tip of an 18-G spinal needle stylet, 2 mm in length, was tapped onto the surface of the AT using a customized device to mark the AT for later radiographic analysis (Fig. 1b).

### Radiographic study

**Fig. 2:**
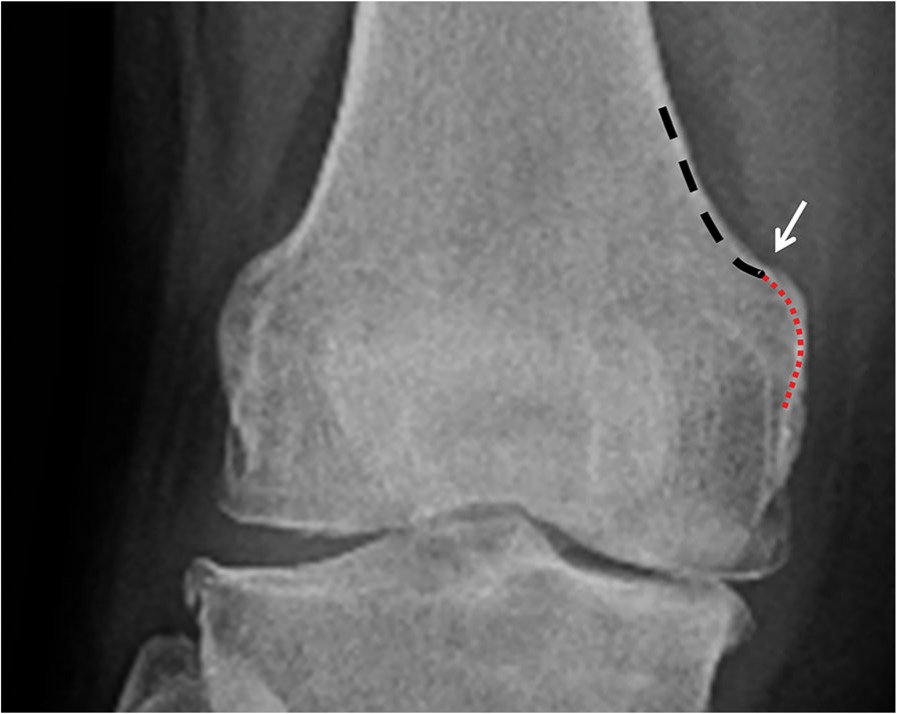
Radiograph showing the inflection point (indicated by the arrow) at the shaft-condylar junction of the distal femur. This point is formed by the juncture of a proximal concave curve (depicted by the coarse arc) and a distal convex curve (depicted by the fine arc)

**Fig. 3:**
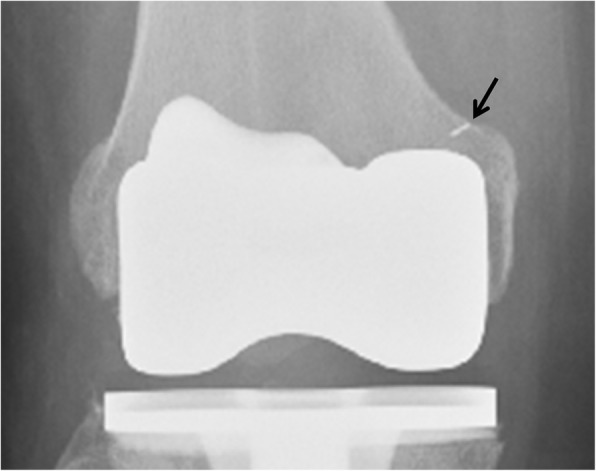
Metal marker on a radiograph and its relationship to the inflection point (arrow). The metal marker was implanted on the adductor tubercle during surgery. On the postoperative radiograph, the marker is located almost at the inflection point

This part of the study was performed on a standard anterior-posterior radiograph of the knee taken after surgery by another surgeon (KTY). First, the location of the metal marker was analyzed to determine where the AT was located on the radiograph. This analysis proceeded with the background knowledge that the metal markers would be located somewhere along the medial border of the femoral shaft-condylar juncture. This silhouette’s configuration was at first concave, and then convex. The concave curve was the radiographic projection of the medial border of the distal femoral shaft or the medial supracondylar ridge. The convex curve was the shadow of the proximal aspect of the medial condyle. The juncture of the two curves made up an inflection point (Fig. 2), which was possibly the location of the AT. With this knowledge, the inflection point was designated as the site reference in this analysis. The vertical distance from the metal marker to the inflection point on each radiograph was measured and recorded (Fig. 3).

**Fig. 4:**
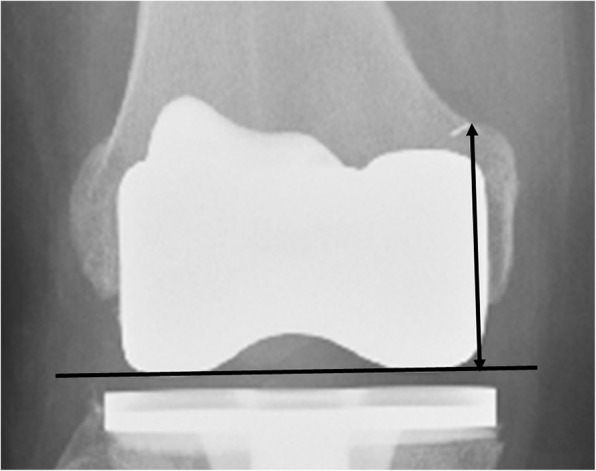
Radiographic joint line distance (RJLD). The distance from the metal marker to the distal condylar surface was defined as the RJLD

Second, on the same postoperative anterior-posterior radiograph, we measured the vertical distance from the marker to the distal femoral joint line (Fig. 4). This measurement was corrected with the radiographic magnification ratio, which was obtained by comparing the dimension between the width of the tibial tray on the radiograph and that of an actual implant. The corrected joint line distance was the radiographic joint line distance (RJLD).

### Statistical analysis

The mean, range, and standard deviation were calculated for the IJLD and RJLD. The intraclass correlation coefficient was adopted to evaluate the inter-rater reliability of the two methods. All statistical analyses were performed using SPSS 17.0 (SPSS Inc., Chicago, IL).

## Results

The location relationship of the metal marker to the inflection point was categorized into the following five groups. Group 1: the marker was located 6 mm proximal to the inflection point and beyond. Group 2: the marker was located between 2 and 6 mm proximal to the inflection point. Group 3: the marker was located within 2 mm either proximal or distal to the inflection point. Group 4: the marker was located between 2 and 6 mm distal to the inflection point. Group 5: the marker was located 6 mm distal to the inflection point and beyond. The results indicated that most markers (53) belonged to group 3 or were located within 2 mm of the inflection point, followed by group 2 (11), group 4 (9), group 5 (3), and group 1 (2).

In the intraoperative study, the mean IJLD was 45 ± 3 mm (range, 38–54 mm) for the 78 knees. In the radiographic study, the mean RJLD was 45 ± 4 mm (range, 38–54 mm) for the same group of knees. The intraclass correlation coefficient adopted to evaluate the inter-rater reliability (0.751) between the two methods was considered high, which provided evidence to support the reliability of measurements between the two approaches.

## Discussion

In this anatomic-radiographic correlation study, we investigated whether the AT could be a radiographic landmark for joint line determination. We showed that the identification of the AT on the radiograph was unambiguous and answered the first study question (the location of the AT on an anterior-posterior radiograph). We also answered the second question by proving that the agreement of the joint line measurement was high between the intraoperative and radiographic measurements.

Clear identification of the AT on the radiograph is the primary prerequisite for performing a joint line distance measurement. The failure of previous investigators to describe the radiographic location of the AT may be attributed to the fact that the AT is a small bony protrusion and that it is shadowed by the prominent anterior bone when a radiograph is taken. Additionally, the AT is not a commonly accessed site in routine orthopedic surgeries; therefore, most surgeons are not familiar with its appearance on radiographs.

Our work indicates that the distribution of AT locations is centered at the inflection point of the radiographic border of the distal femur. In other words, the inflection point is the location of the AT. This result is easy to explain. The AT is a transitional structure between the medial supracondylar ridge and the medial condyle; therefore, it is not surprising that the inflection point, which represents the juncture of these two structures on an X-ray image, is the radiographic location of the AT. This inflection point of the radiographic silhouette is unique and cannot be missed on any knee radiograph. Thus, we designated this inflection point as the AT and performed the relevant measurements.

In addition, even though the AT can be easily identified on radiographs, we must still prove that the linear measurement of the RJLD made on the two-dimensional image equals the spatial measurement of IJLD performed in an operation setting. This inquiry constituted the second part of our analysis. Because the IJLD measurement is the actual measurement and was thoroughly described in our previous study [18], it can be applied as the gold standard technique in this study. When the statistical analysis shows high agreement between the measurement of the IJLD and RJLD for each particular knee, the RJLD can then be verified as a suitable technique.

Once the AT has been proven to be an eligible radiographic landmark, more useful functions can be attributed to it than to other landmarks. Currently, the tibial tubercle, lower pole of the patella, tip of the fibular head, medial epicondyle of the femur, and AT are named “landmarks” for determining the joint line level. However, they are not the same regarding their functional capability. For example, the tibial tubercle, lower pole of the patella, and tip of the fibular head can be identified clearly on radiographs. They have often been used to investigate the joint line status on postoperative radiographs and are considered radiographic landmarks [1, 4, 11]. However, they are rarely used during surgery because they cannot be located or effectively used to determine the joint line position. By contrast, the medial epicondyle can easily be identified during surgery and can be an effective intraoperative landmark [9, 22]. However, it cannot be identified on radiographs clearly and is not regarded as a radiographic landmark.

**Fig. 5:**
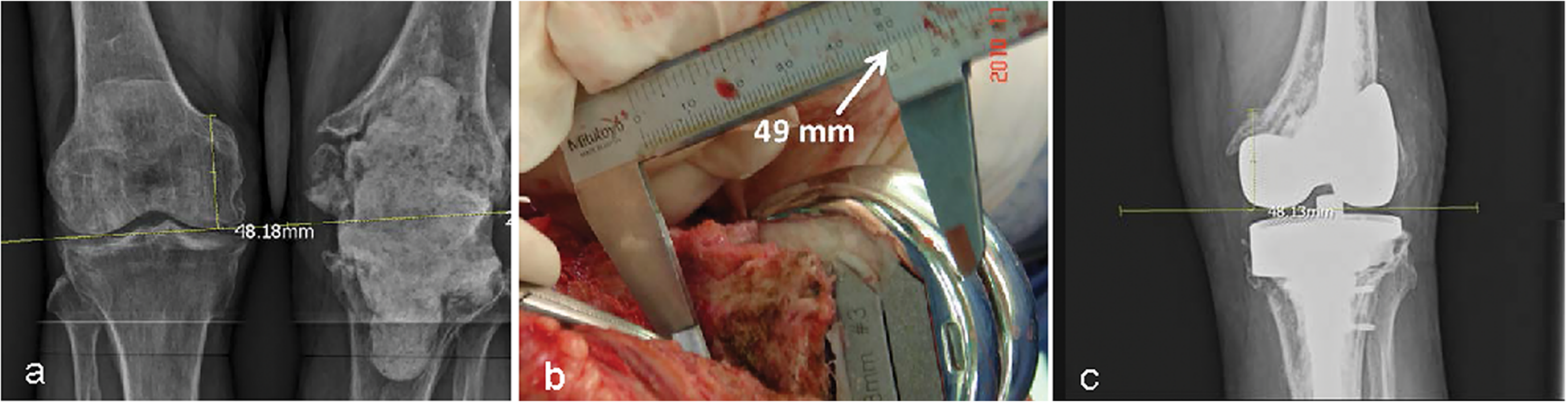
Application of the radiographic joint line distance (RJLD) measurement in revision knee arthroplasty, which is associated with significant bone loss. **a** The RJLD of the contralateral knee measured 48.18 mm on the scaled X-ray image. **b** During operation, an addition of a total of 16-mm thickness augmentation almost restored the planned joint line distance, as was shown by the intraoperative joint line distance measurement. **c** The RJLD (48.13 mm) measured on the postoperative radiograph confirmed the restoration of the joint line position in surgery

In contrast to the aforementioned landmarks, the AT, already considered an ideal intraoperative landmark, was also demonstrated to be an eligible radiographic landmark in this study. Its dual character provides meaningful advantages in managing revision knee arthroplasties with significant bone loss. In this context, for example, the RJLD can be measured on the contralateral knee before surgery to obtain the presumed joint line distance. During surgery, the distal build-up of the femoral condyle would be performed in such a manner that the IJLD would equal the RJLD; after surgery, the result of the joint line reconstruction could be examined with the RJLD measured from the prosthetic joint (Fig. 5). In addition, since the adductor tubercle is located at the most proximal part of the medial condyle, it should be less likely to be influenced by the common complication of revision knee arthroplasties as the discontinuity of the medial condyle from the shaft, which often is caused by loosening of the primary prosthesis. If even the adductor tubercle loses its anatomical bond to the shaft, a tumor prosthesis should be used. Then the joint line position is no longer an important issue in this situation.

This study has several limitations. First, only one orthopedic surgeon measured the IJLD and another measured the RJLD. We did not perform any intra- and interobserver reliability analysis for these two parameters, which inevitably becomes a flaw of the study, although repeated measurements are unlikely to be conducted in a surgical setting. Second, we tried to correct the geographic magnification of the radiographs through the use of a radiographic reference with a known physical dimension. In this case, the tibial tray was the object chosen, and thus, a standard knee radiograph was required to obtain an accurate correction. However, radiographs of suboptimal quality could be avoided, which may influence the accuracy of the correction. Third, judgment of the location of the inflection point is not straightforward; instruction and training are required before a surgeon can master the reading. The last, although interobserver reliability from the clinical study was good, the marginal error of the method as identifying AT was not calculated.

## Conclusion

In summary, we have shown that the AT can be located clearly on an anterior-posterior knee radiograph and that the RJLD, which is based on the AT, is identical to that of the IJLD, which is already a well-developed technique. Both results point to the conclusion that the AT is an eligible radiographic landmark for joint line determination. Thus, the AT becomes the only landmark to possess both intraoperative and radiographic landmark functions. This characteristic makes the AT the best landmark for use in knee arthroplasty associated with significant bone loss.

## Data Availability

The datasets used and/or analyzed during the current study are available from the corresponding author on reasonable request.

## Abbreviations

AT: Adductor tubercle
IJLD: Intraoperative joint line distance
RJLD: Radiographic joint line distance
